# A quick and reliable menthol-induced bleaching protocol for the Caribbean staghorn coral, *Acropora cervicornis*

**DOI:** 10.7717/peerj.20888

**Published:** 2026-03-04

**Authors:** J. Grace Klinges, Marina Villoch Diaz-Mauriño, Roger M. Wilder, Maia C. Erbes, Eleftherios C. Karabelas, Erinn M. Muller, Cory J. Krediet

**Affiliations:** 1 Center for Global Discovery and Conservation Science, Arizona State University, Hilo, Hawai’i, United States of America; 2 Mote Marine Laboratory International Center for Coral Reef Research & Restoration, Summerland Key, FL, United States of America; 3 Department of Biology, Bowdoin College, Brunswick, ME, United States of America; 4 Department of Biological Science, Florida State University, Tallahassee, FL, United States of America; 5 Department of Oceans, Hopkins Marine Station of Stanford University, Pacific Grove, CA, United States of America; 6 Coral Health & Disease Program, Mote Marine Laboratory, Sarasota, FL, United States of America; 7 Department of Marine Science, Eckerd College, St. Petersburg, FL, United States of America

**Keywords:** Coral, Bleaching, Staghorn, Symbiosis, Coral reef, Menthol, Photosynthesis

## Abstract

Corals and dinoflagellate algae form a unique mutualistic symbiosis that provides the energetic and structural foundation for shallow coral reef ecosystems. Despite the long success of this partnership in oligotrophic seas, coral reefs are in decline due to increasing threats from rising seawater temperatures and disease, both of which can lead to bleaching and mortality. In order to better understand the mechanisms that underpin this mutualism, it may be necessary to dismantle the coral-algal symbiosis. Previous studies have experimentally bleached corals using thermal stress, photosynthetic inhibitors (DCMU), and menthol. We compared lab-induced bleaching of staghorn coral *Acropora cervicornis* by menthol treatment to traditional thermal stress. The larger aim was to adapt existing bleaching protocols for this important coral species, providing a guide for future studies. Bleaching in corals treated with menthol or exposed to elevated temperature stress (31°C) was monitored by measuring photosynthetic activity determined by Fv/Fm using pulse-amplitude modulated (PAM) fluorescence and compared to untreated conspecifics. Corals were also monitored for symbiont density and overall health using the CoralWatch Coral Health Chart card throughout the experiment. We found that *A. cervicornis* bleached in response to both menthol treatment and thermal stress, but menthol treatment was more effective at reducing algal symbiont photosynthetic capacity (Fv/Fm) without negatively affecting the health of the coral. Our results indicate that menthol treatment at 0.38 mM rendered staghorn coral aposymbiotic within fourteen days without any visual or physiological damage to the coral. This study provides a simple and effective menthol-bleaching treatment protocol for future studies on staghorn coral.

## Introduction

The co-evolved mutualism between corals and their photosynthetic algal symbionts (family Symbiodiniaceae) provides the foundation for shallow coral reefs in oligotrophic waters. However, one of the greatest threats to coral-reef ecosystems in the present era of global climate change is the increasing frequency of coral bleaching events (Brown, 1997; Douglas, 2003; Lesser, 2007; Hoegh-Guldberg et al., 2017). These bleaching events, frequently caused by high sea-surface temperatures, are characterized by diverse coral physiological cascades that ultimately result in the exocytosis or symbiophagy of algal symbionts or apoptosis of coral cells (Douglas, 2003; Weis, 2008; Helgoe et al., 2024). Because the coral animals rely on photosynthetic products from their algal symbionts for much of their metabolic requirements, the breakdown of the symbiosis can result in coral death (Davy, Allemand & Weis, 2012; Helgoe et al., 2024).

Due to the catastrophic impact bleaching events have had on coral cover and diversity worldwide (Hughes et al., 2017; Sully et al., 2019; Manzello et al., 2025), there is a great need to understand bleaching mechanisms and identify possible interventions to increase bleaching tolerance. The ability of corals to switch algal symbiont communities after natural bleaching events has been well-documented (Cunning, Silverstein & Baker, 2015; Boulotte et al., 2016; Quigley et al., 2022). Recently, researchers have attempted to experimentally manipulate algal symbiont communities in *Acropora* by introducing or enriching thermotolerant strains of Symbiodiniaceae to enhance coral bleaching resistance (Matsuda et al., 2022; Buzzoni, Cunning & Baker, 2023). These efforts include inoculation with stress-tolerant symbionts at various coral life stages, though the persistence of thermally-tolerant symbiont lineages outside of nursery contexts has been inconsistent (Abrego, Van Oppen & Willis, 2009; Karp et al., 2025). In order to better understand the mechanisms of bleaching resistance in corals, it may useful to experimentally separate the members of the holobiont (the coral and all of its associated microorganisms, including endosymbiotic dinoflagellates of the family Symbiodiniaceae). Further, as cryopreservation is increasingly employed to preserve coral lineages, the most effective preservation methods require bleaching corals prior to vitrification due to the differing cryo-permeabilities of algal symbionts and coral tissue (Hagedorn et al., 2010; Hagedorn, Farrell & Carter, 2013; Lager et al., 2023). To functionally disassemble the coral-algal symbiosis, corals can be bleached using temperature stress (either cold shock or elevated temperature, Steen & Muscatine, 1987; Muscatine, Grossman & Doino, 1991; Fitt et al., 2001) or photosynthetic inhibitors (*e.g.*, DCMU, Jones, 2004; Wang et al., 2012; Matthews et al., 2016; Bauer et al., 2025). However, these traditional methods to bleach corals have also led to mortality in corals and sea anemones (Watanabe, Yuyama & Yasumura, 2006; Eakin et al., 2010; Muller, Bartels & Baums, 2018).

The coral species *Acropora cervicornis* was once a dominant reef-building species in the Caribbean, but has been driven to functional extinction in the wild by disease and thermal stress, including the ninth mass coral bleaching event affecting Florida’s Coral Reef in 2023 (Manzello et al., 2025). Despite this, restoration activities continue to utilize this species due to its fast growth and demonstrated success as a restoration species prior to 2023 (Schopmeyer et al., 2017; Ladd, Burkepile & Shantz, 2019; Esplandiu et al., 2024). Based on successful trials of menthol in inducing bleaching in other species of coral and anemones, we elected to compare the use of menthol with more traditional methods (*i.e.,* thermal stress) of inducing bleaching in *A. cervicornis* in an effort to decrease mortality and coral stress during symbiont manipulation. Menthol, a compound found in mint-related plants, has been studied for its ability to trigger stress responses in coral similar to those caused by environmental stressors like temperature fluctuations or light stress (Wang et al., 2012; Matthews et al., 2016). Menthol has primarily been used in invertebrate systems for its anesthetic properties (Moore, 1989; Janes, 2008; Winlow et al., 2018; Yamazaki, Kuwahara & Takahashi, 2024) but recent studies have demonstrated its ability to inhibit photosynthesis (Wang et al., 2017; Clowez et al., 2021) and cause bleaching in corals and anemones (Wang et al., 2012; Matthews et al., 2016; Puntin et al., 2024; Bauer et al., 2025). By exposing corals to menthol, we aimed to simulate bleaching events in a controlled environment and to develop an efficient bleaching protocol for *Acropora cervicornis*, allowing us to study the physiological and molecular responses of corals to bleaching without subjecting them to actual thermal stress. This method has shown promise in aiding our understanding of coral bleaching mechanisms, will help facilitate further research on symbiont manipulation in *A. cervicornis*, and can be immediately integrated into cryopreservation pipelines to maintain biorepositories of this species.

## Materials & Methods

Portions of this text were previously published as part of a preprint (10.1101/2025.09.29.679371). All experiments were conducted at Mote Marine Laboratory’s International Center for Coral Reef Research in Summerland Key, Florida (24°39′41.9″N, 81°27′15.5″W). The use of nursery-grown corals was authorized by the Florida Keys National Marine Sanctuary under Special Activity License SAL-21-2048A and SAL-222406A-SCRP. Replicate fragments of *Acropora cervicornis* genotype ML-31 were collected from Mote Marine Laboratory’s *in situ* nursery in April 2023, and were fragmented into five cm-long ramets and mounted to ceramic plugs (Boston Aqua Farms, Windham, NH, USA). Genotype ML-31 has been well-studied, with information published on its fecundity (Koch et al., 2022b; Koch et al., 2022a), growth rates (Koch et al., 2022b), bleaching susceptibility (Klepac et al., 2024), disease susceptibility (Muller, Bartels & Baums, 2018), and microbiome composition and variability (Klinges et al., 2020; Aguirre et al., 2022; Williams et al., 2022). The distinct genetic identity of this genotype has been confirmed by both 2bRAD and SNPchip. ML-31 has displayed moderate bleaching susceptibility and appears to be more susceptible to acute temperature stress than chronic temperature stress (Klepac et al., 2024). Algal symbiont communities are homogenous across Mote *A. cervicornis*, with *Symbiodinium fitti* strain A3 dominating all genotypes. We selected genotype ML-31 because it is an average performer in response to thermal stress and sufficient biomass could be easily procured due to its high growth rates.

Fragments were allowed to acclimate to *ex situ* conditions for one month prior to experimentation. Corals were housed in 5-gallon aquaria held in temperature-controlled, flow-through seawater raceways (∼170 gallons) with natural locally-sourced seawater from the Atlantic side of the Keys. Sand- and particle-filtered water was fed from header tanks to aquaria at a flow rate of 7.2 L/hr. Aquaria were located outdoors under natural light regimes with the addition of 75% shade cloth from 11:30 am to 2:30 pm daily to maintain PAR at ∼300 µM/s during daylight hours. Five replicate corals were placed in each aquarium, and the egg crate racks holding corals were set to be 18 cm below the surface of the water. Aquaria were distributed between two flow-through seawater raceways (20 aquaria per raceway), which allowed for temperature regulation of individual aquaria. Raceway water was prevented from entering aquaria by maintaining raceway water levels below aquarium height using a standpipe. All corals were fed a standard diet of MicroVore, Zooplanktos-S, and Reef Snow (all Brightwell Aquatics, Fort Payne, AL) twice per week.

For comparison to menthol treatments, 15 coral fragments were bleached using elevated temperature (31.5 °C) and an additional subset was maintained at ambient temperature (27.5 °C) with no menthol treatment as a control. Elevated temperature treatments were performed in 5-gallon aquaria within outdoor raceways under ambient lighting. Temperature was controlled by a boiler and chiller using a dual heat exchanger system connected to header tanks and individual raceways. Header tank pH was stabilized at ∼8.0 by aeration and mixed *via* a venturi pump system. Elevated temperatures were achieved by incrementally increasing raceway temperature by 0.5 °C per day for eight days to reach 31.5 °C. Temperature, pH, dissolved oxygen, and salinity were measured in all raceways using a YSI (YSI Pro Plus, Xylem Inc, Washington, DC, USA) twice a day (8:00am and 12:00pm) three days a week (Monday, Wednesday, and Friday).

Menthol incubation was performed on a total of 15 individual corals for 6 hrs (∼11 am to 5 pm) in aerated aquaria filled with 17L of 0.2 µm filtered seawater. Aquaria were maintained indoors in low-light conditions with ambient light provided from a nearby window (∼100 PAR as measured by Licor Li-1500 and Li-192 underwater quantum sensor) (Lager et al., 2023; Lager et al., 2024). These conditions were selected to evaluate response to menthol alone, as light alone (350 PAR) has been shown to bleach corals (Lager et al., 2024). Temperatures were maintained at 27 °C using 100 W titanium aquarium heaters (Bulk Reef Supply) with Helio temperature controllers and tanks were aerated with an aquarium air pump. Additional water movement was provided using a 30 gph aquarium water pump. Three doses of menthol were tested, as in Wang et al. (2012): 0.19 mM, 0.38 mM, and 0.58 mM (99%; Sigma Aldrich, St. Louis, MO, USA). Menthol was added to aquaria as 20% menthol in ethanol (w/v) (*N* = 5 fragments per dose treatment). Menthol in ethanol was added to aquarium water prior to introduction of corals to allow complete dissolution. Aquarium water and menthol were replaced every other day to maintain water clarity as symbionts were lost. Each day, after 6hr incubation, coral fragments were returned to the holding raceway and held at 27 °C and ambient outdoor light for 17–18 h (overnight) under conditions identical to the control treatment. The fragments were cycled daily between the menthol bath and raceway conditions for two weeks until they were fully bleached (reached the lowest color ranking on a CoralWatch Coral Health Chart, Siebeck, Logan & Marshall, 2008). After two weeks, corals were allowed to recover in raceways for one month to observe continuing impacts of treatment on coral health.

To monitor bleaching progression throughout the menthol or thermal stress treatments, we measured photosynthetic activity as Fv/Fm through pulse-amplitude modulated (PAM) fluorometry using a Junior-PAM (Heinz Walz, Effeltrich, Germany). The Junior-PAM employs a blue LED (pulsed through a fiber optic cable) for pulse modulated excitation light, actinic illumination, and saturation pulse analysis of photosystem II. The fluorometer has a far-red LED for selective excitation of photosystem II, which is needed for determination of F0 (minimum fluorescence yield while dark-adapted) fluorescence. Photosynthetic activity was measured at day 0, 7, and 14 of the menthol treatments and at day 0, 7, 14, and 28 of the elevated temperature treatment. Prior to measurement, corals were dark-adapted for 30 min to capture maximum photochemical efficiency of photosystem II (as Fv/Fm). Measuring light intensity was set to 6, SAT-Pulse Intensity was set to 12, SAT-Pulse width was set to 0.6, and Gain was set to 3. Measurements were taken as three saturation pulses back-to-back in triplicate per coral, selecting different areas of the coral fragment to acquire each measurement. *F*_v_/*F*_m_ was calculated from measured minimum and maximum fluorescence (*F*_o_ and *F*_m_) as (*F*_m_ − *F*_o_)/*F*_m_. Data were averaged within each replicate fragment (three measurements per fragment). Differences in Fv/Fm across timepoints were assessed using a Kruskal-Wallis rank sum test, followed by pairwise Wilcoxon rank sum tests with false discovery rate (FDR) correction for multiple comparisons. Rough assessments of algal symbiont concentrations and coral health were made weekly using a CoralWatch Coral Health Chart (Siebeck, Logan & Marshall, 2008). The CoralWatch Coral Health Chart provides a six-point scale with which changes in coral color can be measured as an indicator of symbiont density. Photographs of each coral individual were taken contemporaneously with coral health card measurements.

All associated code and raw data is available at 10.5281/zenodo.17886195.

## Results

**Figure 1:**
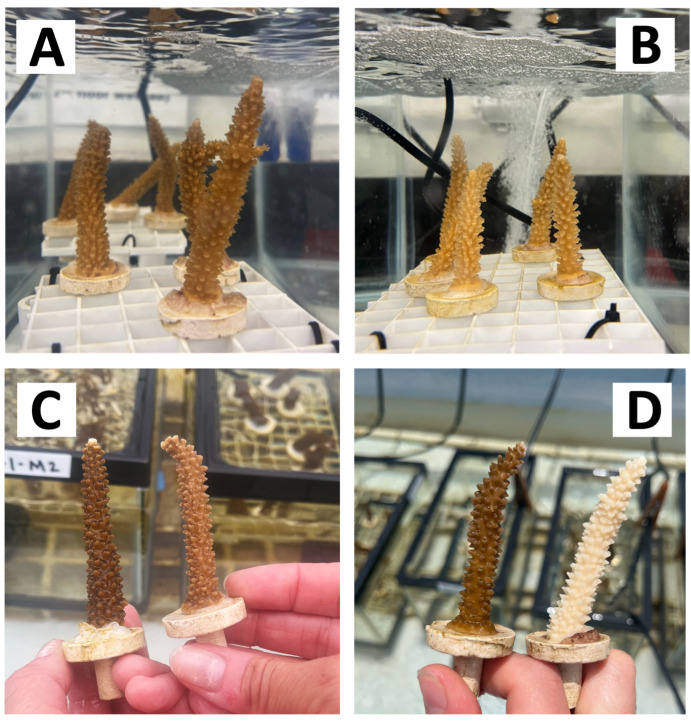
Coral health during menthol treatment. Corals exhibited polyp extension, indicative of good health during menthol bleaching. (A) Corals on the first day of menthol treatment (0.38 mM dose). (B) Corals on the third day of menthol treatment (0.38 mM dose). (C) Untreated coral (left) compared to menthol-treated coral (0.58 mM dose) at 48 h. (D) Untreated coral (left) compared to menthol-treated coral (0.58 mM dose) at 2 weeks.

**Figure 2:**
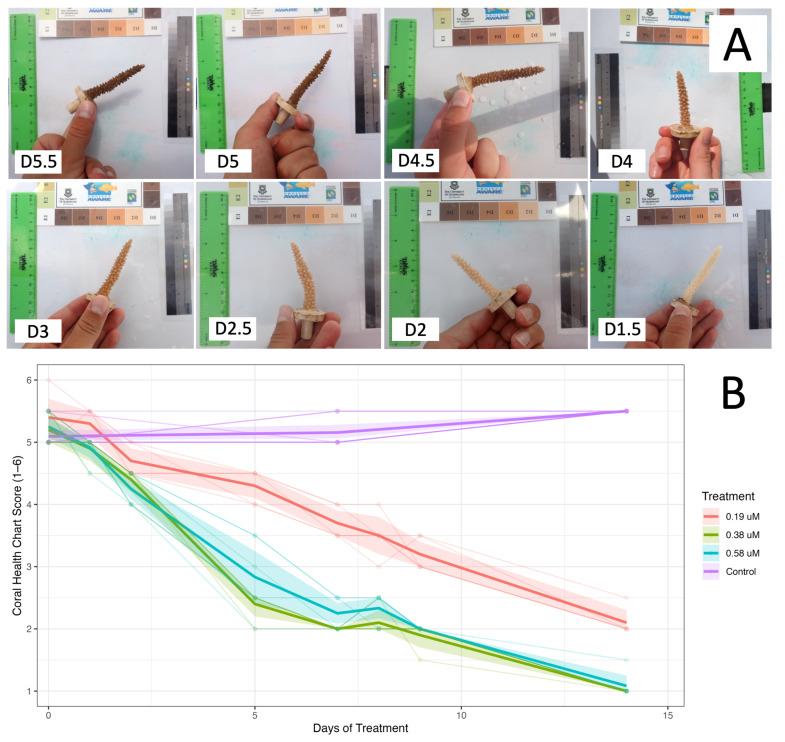
Coral health scores as quantified by CoralWatch chart. (A) The CoralWatch Coral Health Chart was used to estimate coral health scores using a scale from D1-D6. Health scores (1–6) were assigned by visual comparison to a standardized color reference card to ensure consistent scoring across observers and timepoints. Representative images for various scores are shown. (B) Mean health scores (±95% confidence intervals) of genotypes across treatments over time. Points represent observed mean health scores (as determined from CoralWatch Coral Health Chart) for each tank treatment at each sampling date, calculated across all genotypes within a treatment. Error ribbons denote 95% confidence intervals around the treatment means. Individual genotype-level observations (faint lines and points) are shown to illustrate within-treatment variability. See Fig. S5 for smoothed linear regression.

Throughout the experiment, corals exhibited no necrosis across any dose of menthol-treated corals, with corals continuing to feed and exhibiting good polyp extension for the first week of menthol exposure (Fig. 1). Limited to no polyp extension was observed in corals bleached with elevated temperature (31 °C), and tissue necrosis was observed on these corals (Fig. S1). We observed visible differences in algal symbiont density between menthol-treated corals and untreated controls as early as 48 h into treatment (Figs. 1 and 2, Fig. S5). On the third day of treatment, corals dosed with 0.58 mM menthol exhibited retracted tissue and significant mesenterial filament extension (Fig. S2). Corals dosed with 0.38 mM had moderate mesenterial filament extension, while corals dosed with 0.19 mM menthol displayed no mesenterial filaments and polyps were extended (Fig. 1).

**Figure 3:**
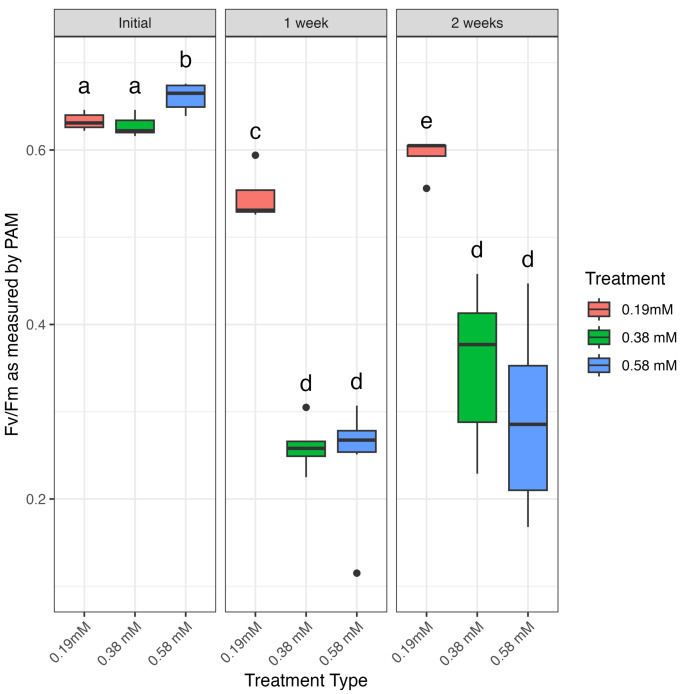
Photosynthetic efficiency during menthol treatment as measured by Junior-PAM. Photosynthetic efficiency measured as Fv/Fm by Junior-PAM, compared between three doses of menthol (0.19 mM, 0.38 mM, and 0.58 mM) prior to treatment and after one and two weeks of menthol treatment. Boxes that share a letter are not significantly different from each other at *a* = 0.05 (Kruskal–Wallis with Pairwise Wilcoxon).

**Figure 4:**
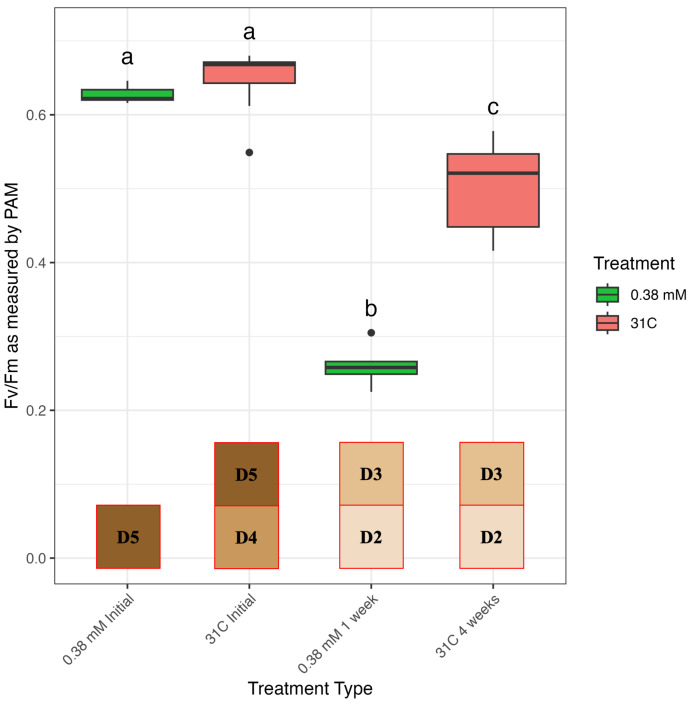
Photosynthetic efficiency compared between menthol and temperature treatment. Fv/Fm as measured by Junior-PAM, comparing corals bleached using 0.38 mM menthol to corals bleached using the more traditional method of thermal stress at 31 °C at day 0 and at the end of treatment (7 days for menthol and 4 weeks for thermal bleaching). Boxes that share a letter are not significantly different from each other at *a* = 0.05 (Kruskal–Wallis with Pairwise Wilcoxon). Also shown for each timepoint across the *x*-axis is the corresponding average coral health score, acquired at the time of Fv/Fm measurements using the CoralWatch Coral Health Chart.

Measurements of photosynthetic efficiency as Fv/Fm were taken for all corals through pulse-amplitude modulated (PAM) fluorometry using a Junior-PAM prior to treatment and at day 7 and day 14 of menthol treatment (Fig. 3) and at day 0, day 7, day 21, and day 28 for corals bleached through elevated temperature (Fig. 4, Fig. S3). High temperature (31 °C) was effective at bleaching corals by three weeks of exposure (Fig. 4, Table S1). The interaction of treatment and timepoint had a significant impact on photosynthetic efficiency of menthol-treated corals as measured by PAM (chi-squared = 42.271, *df* = 8, *p*-value = 1.205e−06). All three doses of menthol, as well as high temperature, were effective in reducing photosynthetic activity from initial/control values of around 0.65 (Table S2), however, bleaching was much more effective at higher doses (0.38 and 0.58 mM) of menthol after only seven days of treatment (Fig. 3, Fig. S4, Table S2). Although Fv/Fm values of corals treated with 0.19 mM menthol were significantly lower than initial values by as early as one week (*p* = 0.0181, Table S2), corals treated with either 0.38 or 0.58 mM menthol had significantly lower Fv/Fm values than both initial values and the 0.19 mM dose (all *p* values < 0.5, Table S2, Fig. 3). Indeed, average Fv/Fm values were significantly lower in menthol-treated corals after only seven days compared to high temperature-treated corals after 28 days (Fig. 4, Table S3). As observed in other studies (Wang et al., 2012; Bauer et al., 2025), we found that the highest dose test (0.58 mM menthol) induced partial fragment mortality that was observed after the two-week experiment period, as corals recovered from treatment. The lowest dose test (0.19 mM menthol) by two weeks significantly reduced Fv/Fm from baseline measurements (*p* = 0.0181, Table S2), and compared to untreated controls (*p* = 0.0393, Table S2), but Fv/Fm at 2 weeks for 0.19 mM menthol was significantly higher than Fv/Fm from both higher menthol doses and from high temperature-bleached corals (all *p* values < 0.05, see Fig. S4, Table S3).

## Discussion

We conclude that menthol treatment is highly effective at bleaching *A. cervicornis* without significant impacts on coral health and suggest 0.38 mM as the optimal dose for this species. Corals treated with 0.38 mM and 0.58 mM menthol showed similar reduction in both Fv/Fm and visible symbiont density (Figs. 2 and 3), and resulted in a lower Fv/Fm than thermally-bleached corals after only one week (Fig. 4). As treatment with 0.38 mM menthol was sufficient to achieve bleaching, and as this treatment with did not cause mortality as has been reported previously in other species (Wang et al., 2012), we suggest this dose be used for bleaching and cryopreservation studies with this species moving forward.

Our results demonstrate that menthol treatment is an effective and reliable method to induce bleaching in staghorn coral, *Acropora cervicornis*, compared to traditional methods using elevated temperature. Menthol treatment yielded significant reduction in algal symbiont densities and significantly reduced Fv/Fm values without compromising the integrity of the coral host. Interestingly, 1 week of menthol bleaching reduced Fv/Fm values further than 4 weeks of temperature bleaching, though both treatments had similar algal symbiont density as visually assessed by CoralWatch card. Further studies could improve on method validation by including symbiont counts or chlorophyll measurements, though the CoralWatch card has been standardized against symbiont density and chlorophyll *a* content (Siebeck, Logan & Marshall, 2008). Temperature bleaching induced total or partial mortality in 93.7% of frags that continued to develop after temperature stress alleviated. Although no mortality was seen during menthol treatment, partial mortality occurred in subsequent weeks after the experiment, impacting 56.3% of frags and which was seemingly independent of treatment. As corals were not re-inoculated with algal symbionts after treatment, this mortality may be related to reduced nutrition from loss of symbionts rather than directly related to treatment. As one week of menthol treatment was sufficient to greatly reduce photosynthetic efficiency, though not to fully bleach corals, a treatment duration between one and two weeks could be sufficient for applications such as cryopreservation that require high survival rates after bleaching. Enhanced methods for recovery of algal symbionts after bleaching have been developed that could further reduce mortality rates after treatment (Morgans et al., 2020; Scharfenstein et al., 2022). Although this study was performed on a well-characterized genotype of *A. cervicornis* that has demonstrated poor-to-average performance in response to thermal stress, menthol sensitivity may vary among genotypes and therefore dosage could be refined for specific critical genotypes through future study.

While menthol treatment clearly provides a reliable means to induce bleaching in *A. cervicornis*, it is important to recognize that the cellular processes underlying menthol- *versus* temperature-induced bleaching are distinct. In vertebrates, menthol is known to bind to Transient Receptor Potential Melastatin 8 (TRPM8), leading to local increases in Ca^2+^ concentrations, and it has been proposed that a similar mechanism may induce Ca^2+^-stimulated exocytosis or symbiophagy of algae in cnidarian systems (Pang & Südhof, 2010; Wang et al., 2012; Dani et al., 2016; Matthews et al., 2016). There is also evidence that menthol disrupts photosynthetic activity in the algal symbionts directly, impairing photosystems I and II and possibly destabilizing thylakoid membranes (Wang et al., 2012; Wang et al., 2017; Clowez et al., 2021), leading to expulsion of symbionts. In contrast, thermal bleaching triggers a more complex host stress response involving reactive oxygen species, protein unfolding, disruption of nutrient and calcium homeostasis, and shifts in host immunity (Weis, 2008; Pinzón et al., 2015; Ruiz-Jones & Palumbi, 2017; Cui et al., 2023; see Helgoe et al., 2024 for review). These mechanistic differences mean that while menthol is highly effective for generating aposymbiotic corals, it may not fully replicate the cascade of physiological changes associated with natural bleaching. Comparative transcriptomic profiling of both algal symbiont and host could help clarify the degree of overlap between these methods and determine the extent to which menthol-induced aposymbiosis represents a true analog for climate-driven bleaching.

As the field of coral restoration continues to build and provide insight into the mechanisms of thermal tolerance, there is an increasing need for reliable methods to experimentally bleach corals and generate aposymbiotic individuals. This has already proven valuable in the coral model system, Aiptasia (Goulet, Cook & Goulet, 2005; Lehnert, Burriesci & Pringle, 2012; Dungan et al., 2020; Roberty et al., 2024) as well as for the cryopreservation of coral microfragments (Lager et al., 2023; Lager et al., 2024). The ability to render and maintain corals in the aposymbiotic state opens opportunities to investigate symbiont establishment and maintenance, host–symbiont recognition, and the functional role of symbiosis under climate stress. Beyond fundamental biology, menthol bleaching provides practical value for cryopreservation workflows, experimental symbiont manipulations, and microbiome or probiotics-based interventions, while reducing the logistical demands and mortality risks associated with prolonged temperature stress. By enabling both mechanistic research and applied restoration strategies, menthol treatment offers a powerful, scalable tool for advancing coral conservation under accelerating global change.

## Conclusions

This study tested the hypothesis that menthol treatment can effectively induce bleaching in *Acropora cervicornis* without compromising coral health. Exposure to 0.38 mM menthol reliably reduced photosynthetic efficiency and symbiont density within two weeks, including greater reduction in Fv/Fm than thermal bleaching while avoiding the acute mortality associated with prolonged heat stress. These findings demonstrate that menthol provides a controlled and reproducible alternative to temperature stress for generating aposymbiotic corals, adding to the experimental toolkit for coral biology and restoration. Nonetheless, delayed partial mortality in some fragments highlights a limitation, as aposymbiotic corals require nutritional support or re-inoculation to ensure long-term survival. Future studies should examine intermediate treatment durations, optimize post-treatment recovery protocols for better integration into cryopreservation pipelines, and compare molecular responses between menthol- and thermally-bleached corals. By establishing a reliable and less-damaging approach to induce aposymbiosis in a threatened reef-builder, this work advances opportunities to investigate coral-algal symbiosis and to streamline cryopreservation methods for endangered acroporid lineages.

##  Supplemental Information

10.7717/peerj.20888/supp-1 Supplemental Information 1 Supplemental materials

## Additional Information and Declarations

### Data Availability

The following information was supplied regarding data availability: All raw PAM files and code for analyzing data are available at GitHub and Zenodo: - https://github.com/graceklinges/menthol-bleaching
- Grace Klinges. (2025). graceklinges/menthol-bleaching: Publication version (v1.0). Zenodo. https://doi.org/10.5281/zenodo.17886196.
