# Supplementary material for: A quick and reliable menthol-induced bleaching protocol for the Caribbean staghorn coral, Acropora cervicornis
Source: PeerJ. 2026 Mar 4;14:e20888. doi: 10.7717/peerj.20888 (PMC12967073; doi:10.7717/peerj.20888)

Supplemental Materials for: A quick and reliable menthol-induced bleaching protocol for the Caribbean staghorn coral, *Acropora cervicornis*

Supplemental Tables

Supplemental Table 1. Adjusted p-values from pairwise Wilcoxon rank sum tests comparing Fv/Fm values across timepoints for temperature-bleached corals (at 31°C) and untreated conspecifics across 4 weeks of treatment and one week of recovery. P-values were corrected for multiple comparisons using the false discovery rate (FDR) method. Significant p-values are bolded and in red.

	Control Initial	31C Initial	31C 1 week	Control 2 weeks	31C 3 weeks	31C 4 weeks	Control 4 weeks
31C Initial	**0.0042**	NA	NA	NA	NA	NA	NA
31C 1 week	**0.0144**	0.4148	NA	NA	NA	NA	NA
Control 2 weeks	0.6061	**0.0144**	0.0584	NA	NA	NA	NA
31C 3 weeks	**2.09E-05**	**9.99E-06**	**9.99E-06**	**1.89E-05**	NA	0.2826	NA
31C 4 weeks	**9.99E-06**	**9.99E-06**	**9.99E-06**	**9.99E-06**	0.2826	NA	NA
Control 4 weeks	0.0759	0.0584	0.1864	0.2517	**9.99E-06**	**9.99E-06**	NA
31C 1 week recovery	**9.99E-06**	**9.99E-06**	**9.99E-06**	**9.99E-06**	0.8209	0.3661	**9.99E-06**

Supplemental Table 2. Adjusted p-values from pairwise Wilcoxon rank sum tests comparing Fv/Fm values across timepoints for menthol-bleached corals and untreated conspecifics at three doses across 2 weeks of treatment. P-values were corrected for multiple comparisons using the false discovery rate (FDR) method. Significant p-values are bolded and in red.

	Control Initial	0.19mM Initial	0.38 mM Initial	0.58 mM Initial	0.19mM 1 week	0.38 mM 1 week	0.58 mM 1 week	Control 2 weeks	0.19mM 2 weeks	0.38 mM 2 weeks
0.19mM Initial	0.2054	NA	NA	NA	NA	NA	NA	NA	NA	NA
0.38 mM Initial	0.6130	0.4496	NA	NA	NA	NA	NA	NA	NA	NA
0.58 mM Initial	**0.0178**	**0.0473**	**0.0313**	NA	NA	NA	NA	NA	NA	NA
0.19mM 1 week	**0.0178**	**0.0181**	**0.0181**	**0.0178**	NA	NA	NA	NA	NA	NA
0.38 mM 1 week	**0.0076**	**0.0181**	**0.0181**	**0.0178**	**0.0181**	NA	NA	NA	NA	NA
0.58 mM 1 week	**0.0064**	**0.0178**	**0.0178**	**0.0178**	**0.0178**	0.6481	NA	NA	NA	NA
Control 2 weeks	0.6130	0.3311	0.5203	**0.0292**	**0.0090**	**0.0076**	**0.0064**	NA	NA	NA

0.19mM 2 weeks	0.1808	**0.0181**	**0.0181**	**0.0178**	**0.0473**	**0.0181**	**0.0178**	**0.0393**	NA	NA
0.38 mM 2 weeks	**0.0076**	**0.0181**	**0.0181**	**0.0178**	**0.0181**	0.1796	0.1544	**0.0076**	**0.0181**	NA
0.58 mM 2 weeks	**0.0064**	**0.0178**	**0.0178**	**0.0178**	**0.0178**	0.6481	0.5183	**0.0064**	**0.0178**	0.3613

Supplemental Table 3. Adjusted p-values from pairwise Wilcoxon rank sum tests comparing Fv/Fm values across timepoints for menthol-bleached corals at three doses across 2 weeks of treatment and temperature-bleached corals (at 31°C) across 4 weeks of treatment. P-values were corrected for multiple comparisons using the false discovery rate (FDR) method. Significant p-values are bolded and in red.

	0.19mM Initial	0.38 mM Initial	0.58 mM Initial	31C Initial	0.19mM 1 week	0.38 mM 1 week	0.58 mM 1 week	31C 1 week	0.19mM 2 weeks	0.38 mM 2 weeks	0.58 mM 2 weeks
0.19mM Initial	NA	NA	NA	NA	NA	NA	NA	NA	NA	NA	NA
0.38 mM Initial	0.43336	NA	NA	NA	NA	NA	NA	NA	NA	NA	NA
0.58 mM Initial	**0.04665**	**0.02987**	NA	NA	NA	NA	NA	NA	NA	NA	NA
31C Initial	0.05853	0.04892	1.00000	NA	NA	NA	NA	NA	NA	NA	NA
0.19mM 1 week	**0.01676**	**0.01676**	**0.01530**	**0.00713**	NA	NA	NA	NA	NA	NA	NA
0.38 mM 1 week	**0.01676**	**0.01676**	**0.01530**	**0.00486**	**0.01676**	NA	NA	NA	NA	NA	NA
0.58 mM 1 week	**0.01530**	**0.01530**	**0.01434**	**0.00380**	**0.01530**	0.65805	NA	NA	NA	NA	NA
31C 1 week	0.10836	0.06377	0.49020	0.42367	**0.00890**	**0.00486**	**0.00380**	NA	NA	NA	NA
0.19mM 2 weeks	**0.01676**	**0.01676**	**0.01530**	**0.01375**	**0.04678**	**0.01676**	**0.01530**	**0.01676**	NA	NA	NA
0.38 mM 2 weeks	**0.01676**	**0.01676**	**0.01530**	**0.00486**	**0.01676**	0.16636	0.14224	**0.00486**	**0.01676**	NA	NA
0.58 mM 2 weeks	**0.01530**	**0.01530**	**0.01434**	**0.00380**	**0.01530**	0.65805	0.49361	**0.00380**	**0.01530**	0.35271	NA
31C 4 weeks	**0.00486**	**0.00486**	**0.00380**	**0.00011**	0.19645	**0.00486**	**0.00380**	**0.00011**	**0.00713**	**0.01375**	**0.00552**

Supplemental Figures

Supplemental Figure 1. Coral bleached by high temperature indicating signs of partial mortality (left) compared to coral bleached by menthol with no signs of partial mortality. Both corals are bleached to a similar degree as measured by CoralWatch coral health chart.

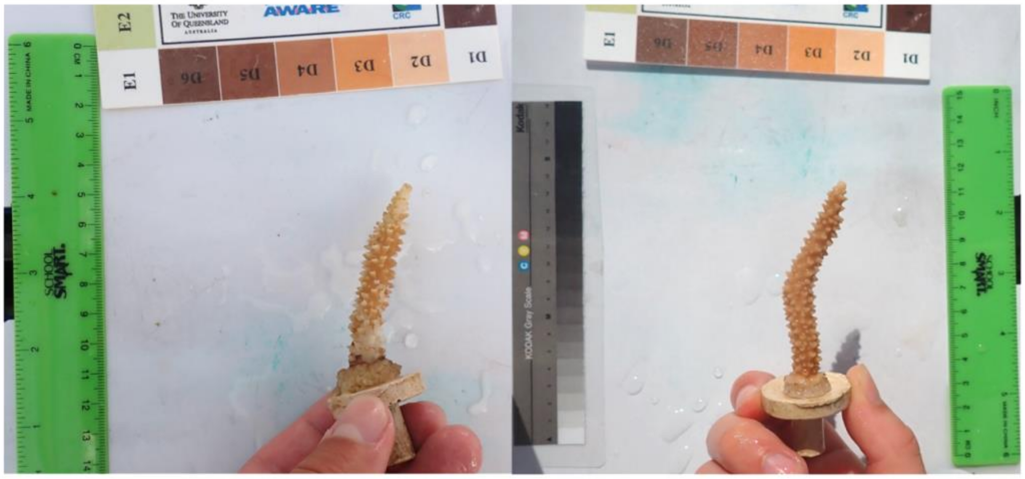

Supplemental Figure 2. Mesenterial filament extension observed on menthol-treated corals. Pale, thread-like strands extend from the mouth of numerous polyps. Tentacles can also be seen, retracted into the coral polyp.

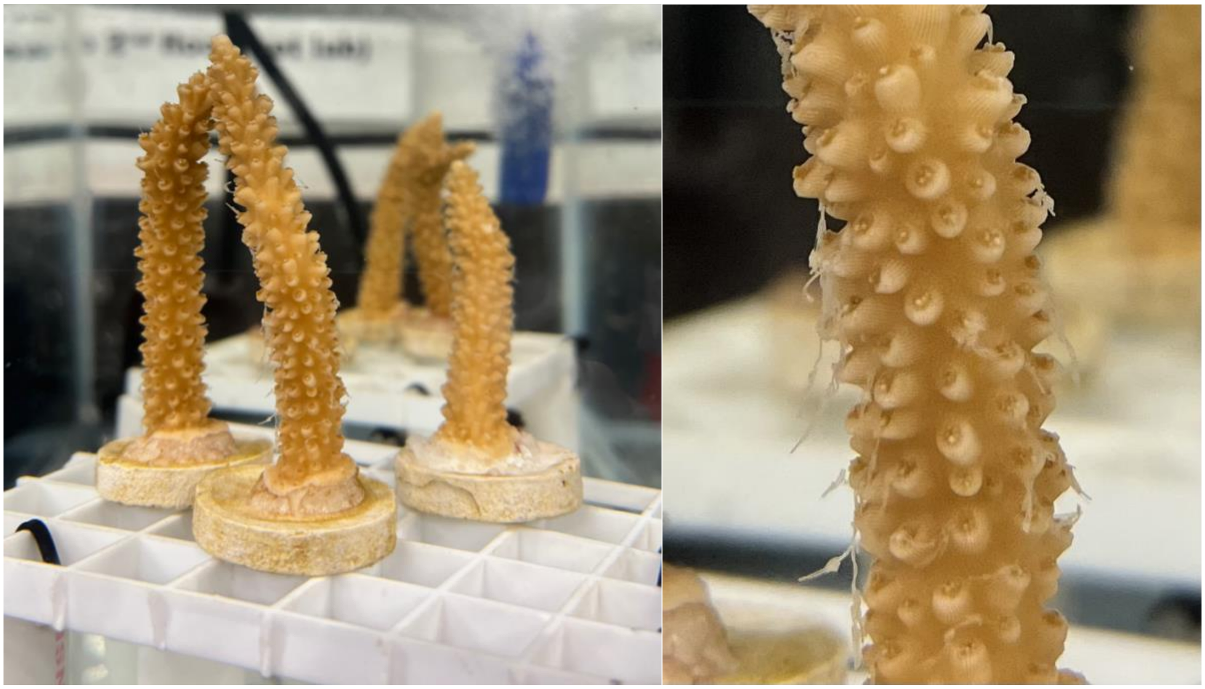

Supplemental Figure 3. Fv/Fm as measured by Junior-PAM, shown for temperature-bleached corals (at 31°C) across 4 weeks of treatment and one week of recovery without controls (A) and with controls (B). Boxes that share a letter are not significantly different from each other at $\alpha = 0.05$ (Kruskall-Wallis with Pairwise Wilcoxon).

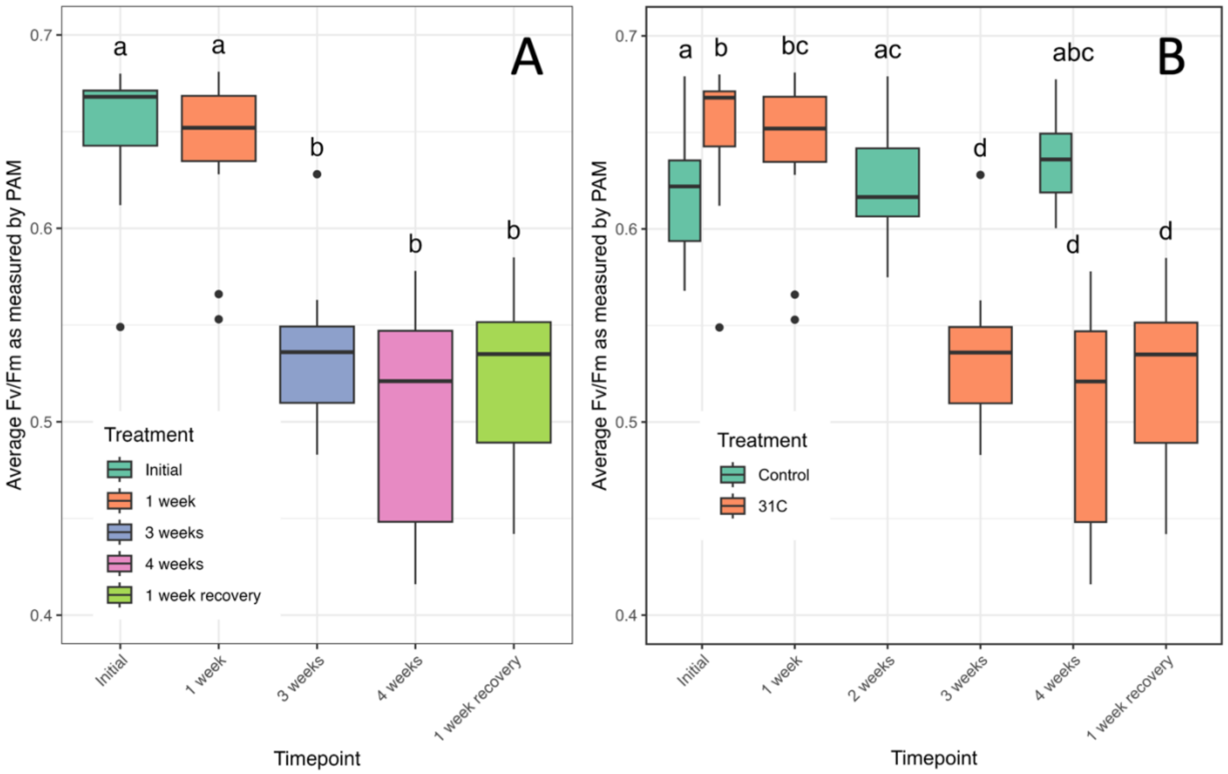

Supplemental Figure 4. Fv/Fm as measured by Junior-PAM at initial, 1 week, and final timepoints for menthol- and temperature-bleached corals. The final timepoint for menthol-bleached corals was at 2 weeks and the final timepoint for temperature-bleached corals was at 4 weeks. Boxes that share a letter are not significantly different from each other at $\alpha = 0.05$ (Kruskal-Wallis with Pairwise Wilcoxon).

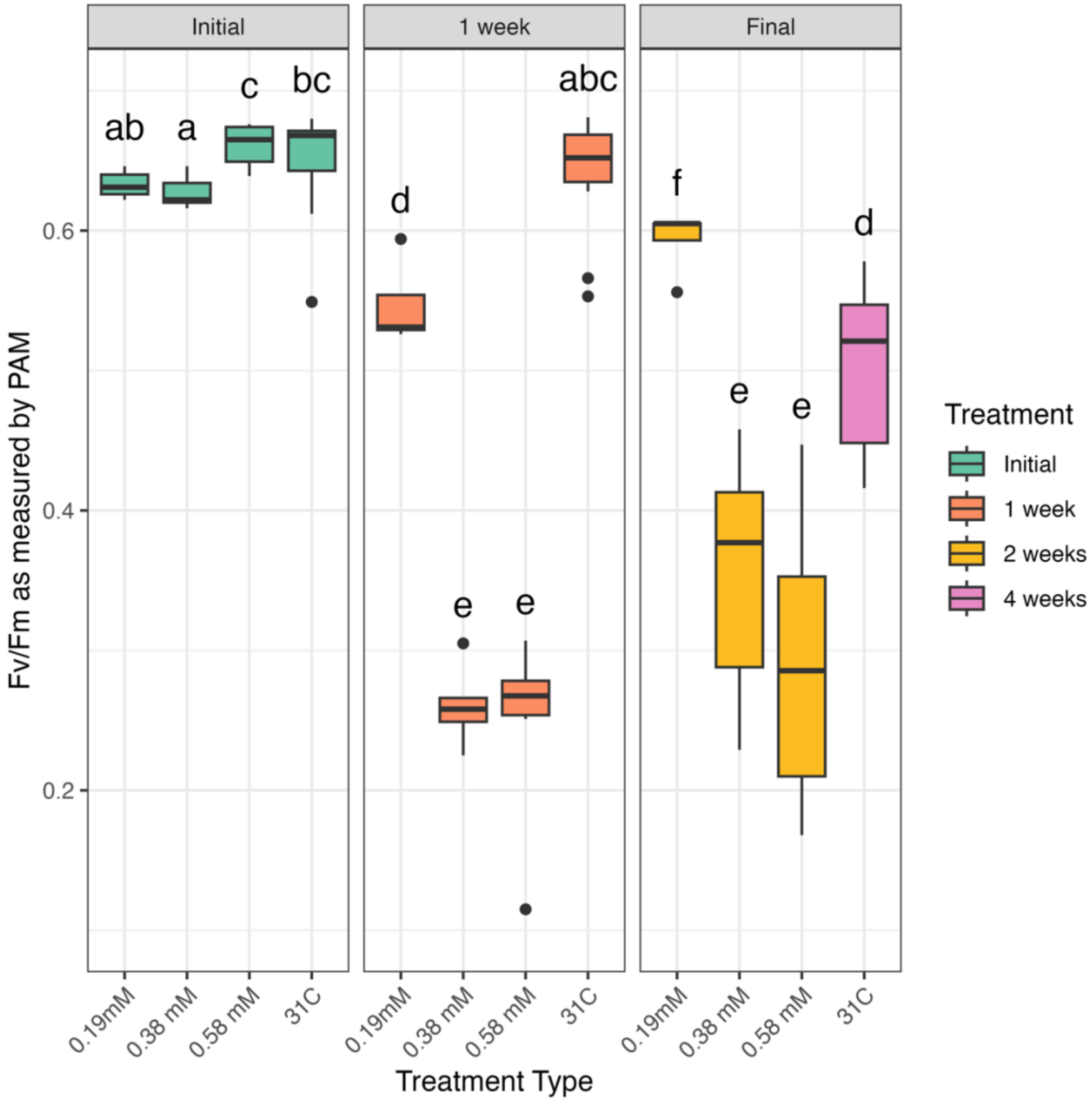

Supplemental Figure 5. Health score trajectories of genotypes under different tank treatments over time, with slopes estimated from generalized linear models. Individual genotypes are shown as faint lines and points to illustrate within-treatment variability. Bold regression lines represent fitted slopes from a generalized linear model of health score as a function of time (days of treatment) and treatment. Reported slope values (lower left inset) indicate the rate of change in health score per day for each treatment. Shaded ribbons represent 95% confidence intervals around model predictions.

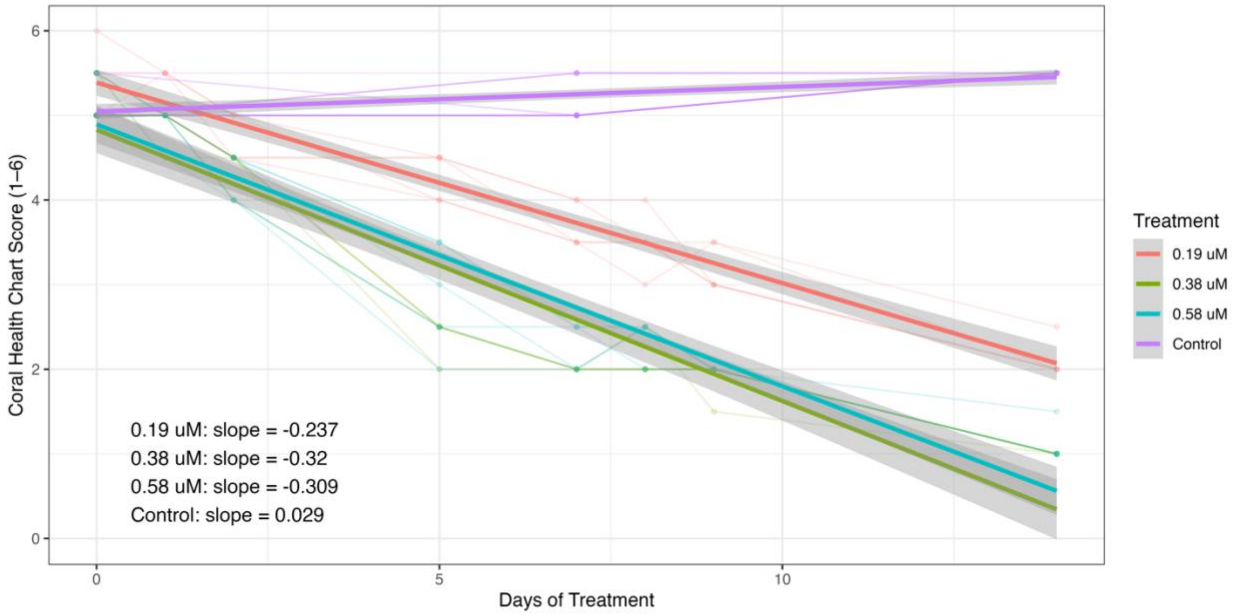

Supplement: Supplemental Information 1 [file peerj-14-20888-s001.pdf]
